# Cost Impact Model of a Novel Multi-mRNA Host Response Assay for Diagnosis and Risk Assessment of Acute Respiratory Tract Infections and Sepsis in the Emergency Department

**DOI:** 10.36469/jheor.2020.12637

**Published:** 2020-04-29

**Authors:** John E. Schneider, Jonathan Romanowsky, Philipp Schuetz, Ivana Stojanovic, Henry K. Cheng, Oliver Liesenfeld, Ljubomir Buturovic, Timothy E. Sweeney

**Affiliations:** 1 Avalon Health Economics, Morristown, NJ; 2 Inflammatix Inc., Burlingame, CA; 3 Medical University Department, Kantonsspital Aarau, Aarau, Switzerland; 4 Department of Endocrinology/Metabolism/Clinical Nutrition, Department of Internal Medicine, Kantonsspital Aarau, Aarau, Switzerland; 5 Medical Faculty, University of Basel, Basel, Switzerland

**Keywords:** Host response, Inflammatix, Cost Impact, Sepsis, Acute Respiratory Tract Infection, Diagnosis, Risk Assessment, Emergency Department

## Abstract

**Background:**

Early identification of acute infections and sepsis remains an unmet medical need. While early detection and initiation of treatment reduces mortality, inappropriate treatment leads to adverse events and the development of antimicrobial resistance. Current diagnostic and prognostic solutions, including procalcitonin, lack required accuracy. A novel blood-based host response test, HostDx™ Sepsis by Inflammatix, Inc., assesses the likelihood of a bacterial infection, the likelihood of a viral infection, and the severity of the condition.

**Objectives:**

We estimated the economic impact of adopting HostDx Sepsis testing among patients with suspected acute respiratory tract infection (ARTI) in the emergency department (ED).

**Methods:**

Our cost impact model estimated costs for adult ED patients with suspected ARTI under the standard of care versus with the adoption of HostDx Sepsis from the perspective of US payers. Included costs were those assumed to be associated with an episode of sepsis diagnosis, management, and treatment. Projected accuracies for test predictions, disease prevalence, and clinical parameters was derived from patient-level meta-analysis data of randomized trials, supplemented with published performance data for HostDx Sepsis. One-way sensitivity analysis was performed on key input parameters.

**Results:**

Compared to standard of care including procalcitonin, the superior test characteristics of HostDx Sepsis resulted in an average cost savings of approximately US$1974 per patient (−31.3%) exclusive of the cost of HostDx Sepsis. Reductions in hospital days (−0.80 days, −36.7%), antibiotic days (−1.49 days, −29.5%), and percent 30-day mortality (−1.67%, −13.64%) were driven by HostDx Sepsis providing fewer “noninformative” moderate risk predictions and more “certain” low- or high-risk predictions compared to standard of care, especially for patients who were not severely ill. These results were robust to changes in key parameters, including disease prevalence.

**Conclusions:**

Our model shows substantial savings associated with introduction of HostDx Sepsis among patients with ARTIs in EDs. These results need confirmation in interventional trials.

## BACKGROUND

Sepsis hospitalizations are one of the most frequent and most expensive conditions faced by US healthcare systems and payers.^1^,^2^ Over 1 million cases of sepsis are reported annually in the US,^3^ of which more than 260 000 present in emergency departments (EDs), accounting for US$15–$27 billion in healthcare costs.^4^–^8^ ICU-based estimates of sepsis incidence in the US ranges from 149 to 367 cases per 100 000 people per year.^9^ EDs account for 500 000 sepsis cases per year, meaning roughly two-thirds of all septic patients entering the hospital through the ED.^9^ Hospital mortality in all patients suspected of sepsis is 4.1%, rising to 28% in those with septic shock.^10^ The incidence of sepsis continues to increase with the aging population, causing an increase in annual mortality rates. Repeated findings have shown that patients with bacterial septic shock have a 7% to 8% increase in mortality for each hour of delay in antibiotics administration, highlighting the need for early and accurate diagnosis and treatment.^11^,^12^

Acute respiratory tract infections (ARTIs) are one of the leading causes of adult hospitalizations, sepsis, and death worldwide, and are also associated with the overutilization of antibiotics.^13^ ARTIs account for 10% of all ambulatory visits and 44% of all antibiotic prescriptions in the US.^14^ Although about 65% of ARTIs are viral,^15^,^16^ antibiotics are prescribed in more than 60% of cases,^15^,^17^ contributing to overuse of antibiotics and increasing antibiotic resistance.^18^–^23^ Antibiotic treatment is associate with a substantial rate of adverse events.^24^,^25^

Both the Surviving Sepsis Campaign guidelines and the Center for Medicare & Medicaid Services (CMS) Sepsis Core Measure (SEP-1) bundle can almost entirely be reduced to two main treatments: (1) source control and antimicrobial therapy to fight an underlying infection and (2) supportive care to maintain physiology. In general, the components can also be split into two separate but complementary axes, namely: (1) whether there is an infection and (2) how severe the underlying condition is. These two separate questions have distinct, but linked, clinical actions. For example, a patient with an acute bacterial infection needs antibiotics. However, the antibiotics chosen for a non-severe acute infection may be narrow oral antibiotics, whereas a patient with a greater risk of organ dysfunction/sepsis may need broad-spectrum parenteral antibiotics. Similarly, patients with non-severe acute infections may not require admission while higher risk cases could be kept in the ED for observation or admitted to a general or ICU ward. Other than lactate, almost all *in vitro* diagnostics for sepsis primarily lead to clinical actions regarding whether and how to properly attain source control and treat with antimicrobials. Modern guidelines often focus on initiating antibiotics within 60 minutes of presentation, and as a result, only diagnostics with a turnaround time substantially shorter than 60 minutes are useful in initial workflow.

Current laboratory tools are inadequate for the diagnosis and prognosis of patients with ARTI and/or sepsis. In particular, culturing bacterial or viral pathogens cannot rule out an infection outside the sampled tissue. Molecular pathogen panels for the detection of respiratory infections are fast and sensitive, but (1) they are confounded by the presence of contaminant and commensal organisms,^26^ (2) they can only detect a defined number of pathogens,^27^ and (3) are expensive as screening tests.

Procalcitonin is the biomarker most extensively studied for its diagnostic and prognostic abilities. In protocol-driven studies, procalcitonin testing during infection treatment reduces antibiotics days and length of stay.^28^–^30^ In addition, the most recent long-term patient-level study by Schuetz et al. found a relative reduction in mortality rate of 1% (absolute reduction of 9%) for acute respiratory infection patients when care was guided with procalcitonin.^13^ However, procalcitonin can also be elevated in patients with non-infectious conditions, such as heatstroke, trauma, surgery, and others.^31^,^32^ Because of these limitations and concerns regarding test accuracy limitations, procalcitonin often does not change clinician behavior.^29^,^33^ For instance, a recent large multicenter US study showed no change in prescribing behavior in an intention-to-treat analysis.^34^

HostDx™ Sepsis (Inflammatix, Inc., Burlingame, CA) is a novel, blood-based 29 target host mRNA test with an advanced proprietary algorithm to inform on (a) the likelihood of a bacterial infection, (b) the likelihood of a viral infection and (c) the severity of the condition (likelihood of mortality).^35^–^37^ The test is designed for use in emergency rooms, urgent care clinics, and inpatient settings.

To conduct an economic evaluation of HostDx Sepsis testing versus standard of care we built a cost impact model based on published data. Our results show substantial savings associated with HostDx Sepsis protocols of ARTI across common US treatment settings mainly by direct reduction of unnecessary hospitalization. These results are robust to changes in key parameters, and the savings can be achieved without any negative impact on treatment outcomes.

## METHODS

We built a deterministic decision-analytic cost impact model to estimate costs for 1000 adult patients with suspected ARTIs presenting to the ED under two scenarios: (1) a base case scenario with standard of care using procalcitonin and (2) a scenario where procalcitonin is replaced with HostDx Sepsis for each patient upon initial presentation. For each scenario, our model estimated episode of care costs, which encompasses the costs of treatment, hospitalization, outpatient visits, and medications. All costs were estimated from the perspective of US payers. In addition to costs, the primary model estimated outcomes including hospital length of stay (LOS), days of antibiotic utilization (antibiotic days), and 30-day mortality.

### HostDx Sepsis

HostDx Sepsis is a novel, blood-based host response test that measures expression of 29 human host mRNAs and interprets them with an advanced proprietary machine learning algorithm to output the likelihoods of bacterial infection, viral infection, and disease severity (30-day mortality). Each result is provided as a numerical score that falls into one of four interpretation bands (very unlikely, unlikely, possible, very likely). Test performance for the three result readouts has been published based on analysis of heterogeneous patient cohorts.^35^–^37^

### Potential Diagnostic Results and Suggested Clinical Actions

**Figure 1:**
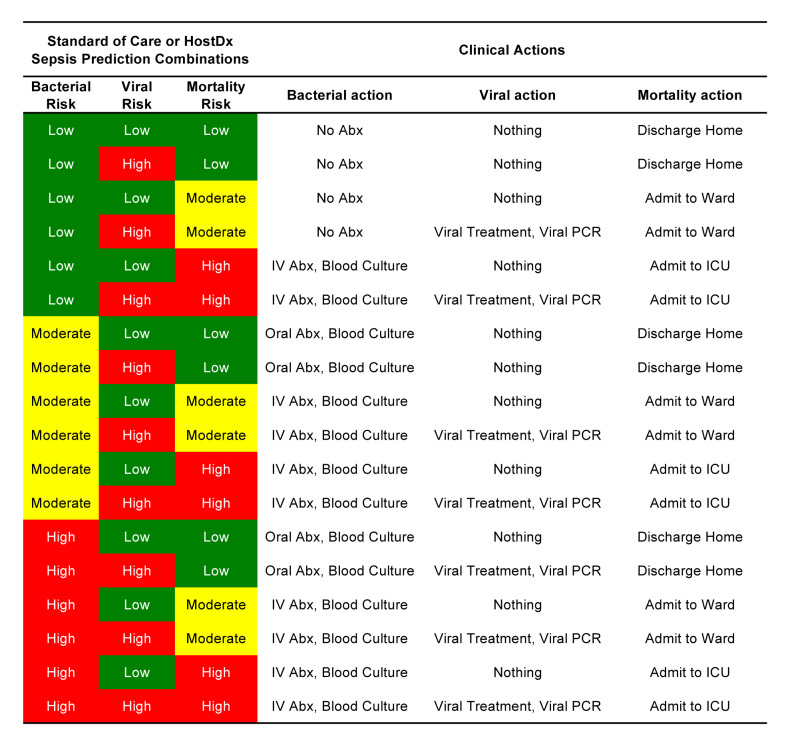
Suggested Clinical Actions for All Combinations of Standard of Care (with Procalcitonin) or HostDx Sepsis Predictions

To simplify our model, only three likelihood bands (low, moderate, and high) were modeled for the risk of a bacterial infection and the risk of 30-day mortality, and only two bands (low or high) were modeled for the viral readout. Two bands were used for the viral diagnostic because we only had a binary action to assign (test and treat if positive). Combining the three individual readouts for bacterial, viral and mortality risks there were a total of 3*2*3=18 potential combinations of bacterial-viral-mortality risk predictions. We then assigned each combination of predictions a clinical action appropriate for ARTI patients in the ED based on assumptions informed by input from key opinion leaders (Figure 1). Critically, we assumed that physicians would always follow these patient management actions for the given standard of care or HostDx Sepsis results, regardless of whether the ground truth aligned with the predictions or not.

### Modeling Outcomes

**Figure 2:**
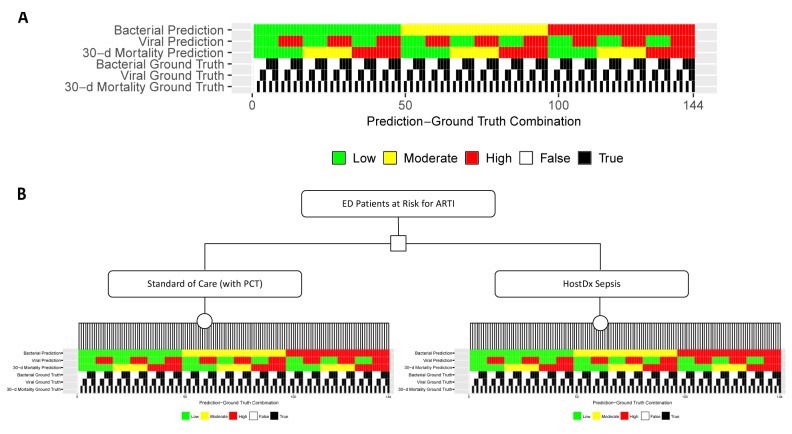
Decision Tree for ED Patients with Suspected ARTI (A) All 144 possible combinations of bacterial, viral, and 30-day mortality prediction results and corresponding ground truths. (B) Decision tree for the cost impact model. Probabilities for patients being placed into each prediction-ground truth combination for each scenario were generated from simulations based on test performance and disease prevalence data.

There are two ground truth states (true or false) for each of the three risk areas, so 2*2*2=8 ground truth states overall (eg, a patient could in fact have a bacterial infection, no viral infection, and a high risk of 30-day mortality). Thus for each of the 18 possible risk prediction combinations there are a total of 8*18=144 possible prediction-ground truth combinations (Figure 2a). We generated clinical outcomes for each prediction-ground truth combination based on clinical data from literature and assumptions (Table 1). For example, when a bacterial infection patient with high risk of 30-day mortality had low-risk predictions for bacterial, viral, and mortality (and so was discharged home without antibiotics), the clinical outcome was a 7-day hospital readmission. Supplementary Material provides a complete list of outcomes for each prediction-ground truth combination.

### Costs

The costs included in the model were those assumed to be associated with an episode of sepsis diagnosis, management and treatment. Costs for each component of suggested clinical actions and clinical outcomes (eg, IV antibiotics, blood culture, hospital readmission) were estimated from literature or assumptions (Table 2). We then calculated the perpatient costs of each prediction-ground truth combination. Since the cost of HostDx Sepsis has not been established as of today, we did not include the cost of HostDx Sepsis into our model.

### Placement of Patients into Prediction-Ground Truth Combination Groups

The performance (AUROCs) of standard of care and HostDx Sepsis tests were derived from clinical studies. We assumed AUROCs for bacterial, viral, and mortality prediction of 0.8, 0.8, and 0.78 in the base case, and 0.85, 0.9, and 0.88 in the HostDx Sepsis case (Table 1).

We built models to test how improved accuracy may be used to make a correct decision in a greater number of patients. To do this, we first simulated ideal receiver operating characteristic curves at the stated AUROCs and broke the curves into bands at preset target likelihood ratios (low-band LR 0.1, high-band LR 10) which roughly correspond to a low-band sensitivity of 93% to 95% and a high-band specificity of 95% to 97%. The same likelihood cutoffs/targets were used for each of the bacterial, viral, and mortality scores. Because the interpretation bands used preset LR targets, for a higher AUROC, more patients were assigned to actionable “high risk” or “low risk” prediction bands. In other words, a more accurate test placed more patients into the “correct” actionable band.

Taking the probability of a case being in a given prediction band, we then multiplied through assumed case prevalence (Table 1) to arrive at expected patient assignments for each of the 144 prediction-truth combinations. This was done for the AUROC assumptions of the base case, and then again for the AUROC assumptions of the HostDx Sepsis test. All of the predictive modeling was accomplished with custom code written in R.

### Cost Calculations and Sensitivity Analysis

The per-patient cost for each prediction-ground truth combination was multiplied by the number of assigned patients to yield final estimates of the total expected costs and clinical outcomes for both the base and HostDx Sepsis scenarios. Finally, we conducted extensive one-way deterministic sensitivity analyses to characterize the robustness of the model on key parameters including diagnostic accuracy, prevalence, cost, and clinical outcomes. With the exception of the custom R script written to place patients into prediction-ground truth combination groups, all aspects of the model was coded in Microsoft Excel.

## RESULTS

### Overall Outcomes

We built a model of diagnostic and prognostic testing of ARTI patients in an ED as described for a standard-of-care (with procalcitonin) base case and a case with the introduction of HostDx Sepsis. Projected accuracies for test predictions, disease prevalence, and clinical parameters were derived from published data and supplemented with internal independent data from Inflammatix. We assumed the perspective of a payer and modeled costs for a cohort of simulated ED patients at risk for ARTI.

On average, using HostDx Sepsis resulted in 0.8 fewer hospital days, 1.5 fewer days on antibiotics, a 1.7% absolute reduction in 30-day mortality, and an expected cost savings of US$1974 per patient compared to standard of care, exclusive of the cost of HostDx Sepsis (Table 3). These reductions correspond to 36.7%, 29.5%, 13.64%, and 31.3% reductions in hospital days, antibiotic days, 30-day mortality and per-patient costs, respectively. For a cohort of 1000 at-risk patients, the potential savings are approximately US$2 million.

### Outcomes by Infection Status and Mortality Ground Truth

Further segmenting these results by ground truth patient characteristics (Table 4) shows the greatest cost savings are generated from a reduction of hospital days among non-severe patients. A substantial reduction in antibiotics days was found for nearly all patient categories. The biggest reductions in antibiotic days was observed for patients that were nonbacterial, non-severe, and either virally infected or non-virally infected. Of interest, even patients that were bacterial but non-severe showed marked reductions in antibiotic days, likely because treatment duration was reduced. An overall increase in ICU days (coupled with mortality reduction) is projected for severe patients, which can be explained by earlier appropriate ICU admissions leading to lower mortality.

### Outcomes by All Possible Base Case/HostDx Sepsis Predictions and Ground Truth Combinations

**Figure 3:**
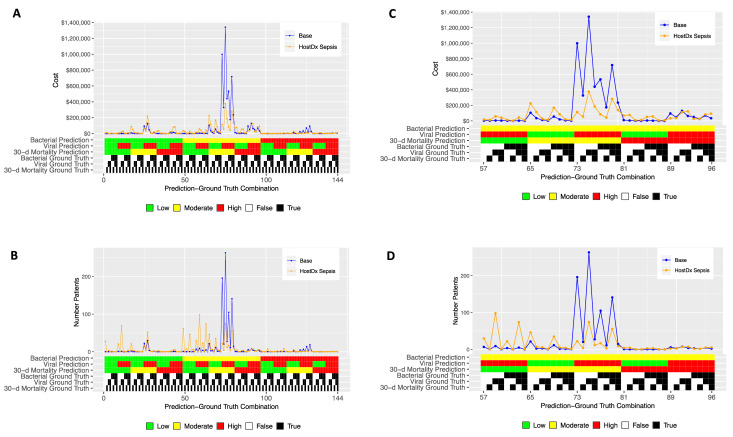
HostDx Sepsis Changes Proportion of Patients in Each Prediction-Ground Truth Combination, Resulting in Cost Savings Estimated costs (A) and number of patients (B) were plotted across all 144 possible combinations of predictions and ground truth for the 1000-patient cohort in both base and HostDx Sepsis cases. Magnified views are shown in C and D.

Next, we segmented estimated costs and number of patients by all 144 possible combinations of prediction and ground truth for the base and HostDx Sepsis cases (Figure 3). The superior performance (higher modeled AUROCs) of HostDx Sepsis pushed relatively more patients into the “actionable” bands at LR 0.1 or LR 10 (low or high, respectively). Aggregate cost savings were driven largely by HostDx Sepsis reassigning patients from “noninformative” predictions of moderate risk for bacterial infections and mortality to actionable predictions of low or high risk.

### Sensitivity Analysis

**Figure 4:**
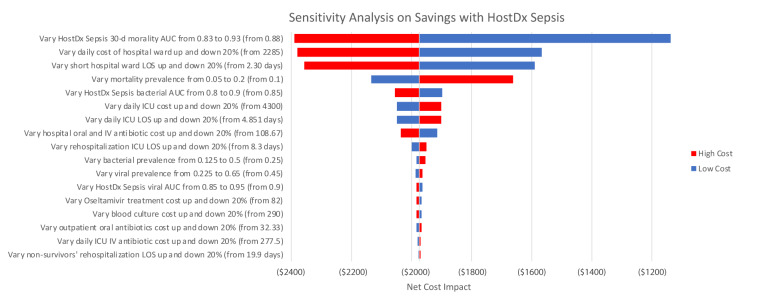
Sensitivity Analysis for Cost Savings of Key Input Variables One-way deterministic sensitivity analysis was performed on key input variables. Most clinical outcomes and cost parameters tested were varied by 20% in each direction. Test accuracies and prevalence parameters were varied up and down based on ranges derived from literature. Red and blue bars indicate the net cost impact if the model was rerun with high-level and low-level estimates of the corresponding parameter, respectively.

We performed one-way deterministic sensitivity analysis on key input parameters. Clinical outcomes and cost parameters tested were varied by 20% in each direction. Test accuracies and prevalence parameters were varied up and down using different ranges based on literature and assumptions (Figure 4).

The HostDx Sepsis scenario was dominant as it generated cost savings for all one-way sensitivity analysis scenarios. The net cost impact results were most sensitive to daily hospital ward costs and hospital ward LOS. A 20% increase in either parameter resulted in a net savings decline of roughly US$400. The next most influential parameters (in descending order) were hospital ICU LOS, ICU cost per day, antibiotic costs and ICU LOS after rehospitalization. Changes in these parameters resulted in minor effects on the overall findings (mostly less than +/− US$100).

## DISCUSSION

In this study we constructed a cost impact model to estimate the cost impact associated with introducing HostDx Sepsis, a novel blood-based host response test that assesses the likelihood of a bacterial infection, the likelihood of a viral infection, and the severity of the condition. The key finding of this study is that introducing HostDx Sepsis results in net expected savings of approximately US$2000 per suspected ARTI patient in the ED, exclusive of the cost of HostDx Sepsis.

Our study showed that the superior performance characteristics of HostDx Sepsis compared to standard of care with procalcitonin allows for a 36.7% reduction in hospital LOS. HostDx Sepsis accomplishes this by reducing the proportion of patients with uncertain “moderate risk” predictions, thus allowing for patients to receive the appropriate level of care to reduce unnecessary days in hospital, freeing up hospital resources. This decrease in hospital LOS was the primary driver of the approximately US$2000 in cost savings per patient we calculated. Considering the average hospital sees 750 ARTI patients in the ED each year,^38^ we estimate the average hospital can save US$1.5 million per year, exclusive of the cost of HostDx Sepsis.

Since the HostDx Sepsis cost and reimbursement rates are not yet known and different for each patient scenario, we did not include them in our model. However, we expect that HostDx Sepsis may be eligible for US$525.81 in CMS reimbursements among CMS patients who are seen in the ED and then directly discharged, This figure is the sum of US$416.78 for 12–25 RNA Taqman probe test (CPT code 87507) and half of the US$218.06 for 6–11 RNA Taqman probe test (CPT code 87506), under 2020 rates (see https://www.cms.gov/Medicare/Medicare-Fee-for-Service-Payment/ClinicalLabFeeSched). Reimbursements from CMS and other payers can help generate revenue for hospital labs which administer and run the tests. On the other hand, ED ARTI patients who are admitted to the hospital will only receive a bundled payment to cover all costs of diagnosis and treatment. The cost savings from HostDx Sepsis can be especially valuable for hospitals under these bundled payment scenarios. Thus, if the cost of HostDx Sepsis to a payer (or hospital) is less than the CMS reimbursement rate of US$525.81, we expect that introducing HostDx Sepsis can result in an average cost savings of at least US$1448.19 per patient.

Additionally, our model estimates that HostDx Sepsis will result in a substantial reduction in antibiotic treatment days. While this reduction may not drive down overall costs from the perspective of the US payer, it can empower hospitals in their goals toward antimicrobial stewardship. Infections caused by resistant bacteria can lead to up to two-fold higher rates of adverse outcomes compared with similar infections caused by susceptible strains.^39^ Reducing the amount of antimicrobial treatment will also reduce the rate of serious adverse effects and *Clostridium difficile* infection among ARTI patients, potentially resulting in added cost savings to payers that are not included in our model.

### Limitations

The study has several limitations. First, the model is a simulation based on the differential probability of certain events at each band or test outcome, and the bands are set at high required stringency (LR=0.1 and LR=10 for low band and high band, respectively). Calculations of expected costs are based on mean values obtained from the literature, and for each parameter there is uncertainty. In the sensitivity analysis, we attempt to assess the importance of this uncertainty by simulating model outcomes under a variety of alternative (but plausible) levels of key input parameters. However, this sensitivity analysis may not encompass all the clinical scenarios observed in practice. Second, the model lacks real-world data. Prospective clinical trials for HostDx Sepsis are currently being conducted and will allow us to update the model with real-world data. Also, while the actual HostDx Sepsis test will split the component scores into four risk bands (very low, low, moderate, and high), only two to three are modeled here, for simplicity in describing clinical actions. In addition, treatment assumptions for each of our prediction-ground truth combinations were based on primarily on key-opinion-leader input, with some level of confirmation from published literature. Again, as is the case with the aforementioned limitation, reliance on these data sources may not accurately reflect all of the clinical scenarios observed in practice.

Surprisingly, our model showed an overall increase in ICU days across all patients. This is due to a limitation in how we simulated ICU transfers given the 30-day mortality risk AUROC data we used for the standard of care arm. As built, the model underestimates the number of patients that would be “ruled-in” as high-severity patients needing ICU care under standard of care. Regardless of this technical limitation, we showed that HostDx Sepsis can lead to (appropriate) increases in ICU days for severe patients who truly needed the care, leading to decreased mortality. Moreover, the overall decrease in hospital days more than made up for the slight overall increase in ICU days, generating cost savings overall. Future models with real-world data will be able to overcome these technical limitations.

For both the base case with procalcitonin and HostDx Sepsis, our model assumes that physicians will have trust in test results and follow all treatment guidelines for standard of care with procalcitonin and HostDx Sepsis results. In real clinical practice, we expect that physicians will not always adhere to treatment guidelines. For example, in the ProACT clinical trial of procalcitonin for lower respiratory tract infections, Huang et al. showed that physicians deviated from procalcitonin guidelines in 72.9% of patients despite defined trainings.^40^

Finally, this model only addressed ARTIs. The HostDx Sepsis test is designed to work across other infection types including abdominal, urinary tract, and skin and soft tissue infections. The current model does not consider the potential benefits of the test’s routine use in these indications.

## CONCLUSION

Patients with suspected ARTI are a high clinical burden. Current standard of care lacks rapid, accurate diagnostic tools, and is characterized by high rates of unnecessary antibiotic prescribing, high hospital resource utilization, and economic inefficiencies. The novel HostDx Sepsis test is estimated to substantially reduce costs, improve treatment and triaging decision-making, and help hospitals achieve antimicrobial stewardship goals. Further studies, including interventional studies, are necessary to confirm these results.

## Supplementary Information



## Figures and Tables

**Table 1:** Baseline Epidemiology and Clinical Parameter Values

Epidemiology & Clinical Parameters	Base Value	Source
Prevalence		
Bacterial infection only	0.25	Assumption
Viral infection only	0.45	Assumption
Bacterial-viral co-infection	0.10	Assumption
No infection	0.20	Assumption
Mortality risk	0.10	Assumption
Test Accuracy		
Base case		
Bacterial AUC	0.80	41
Viral AUC	0.80	42,43
Mortality AUC	0.78	4
HostDx Sepsis		
Bacterial AUC	0.85	35
Viral AUC	0.90	35
Mortality AUC	0.88	37
Clinical Outcomes, Initial Diagnosis Admissions		
Antibiotic days: ED	3.18	30
Antibiotic days: Hospital ward	5.02	30
Antibiotic days: ICU	6.86	30
Length of stay: short hospital ward	1.77	44
Length of stay: ICU	4.85	44
Mortality: septic patients in ICU (viral)	23.0%	45
Mortality: reduction if timely admit	30.0%	46–50
Mortality: ARTI patients	10.0%	50–52
Mortality: viral infection	6.7%	53
Clinical Outcomes, Rehospitalization Admissions		
ICU length of stay	8.30	54,55
Non-survivors length of stay	19.90	56

**Table 2:** Baseline Cost Parameter Values

Cost Parameters	Base Value (US$)	Source
PCR viral testing	$129.00	57–60
Blood culture testing	$290.00	61
Oseltamivir (episode of care treatment)	$82.00	62
Antibiotics cost (oral) outpatient	$32.33	63
Antibiotics cost (oral and IV) hospital setting	$108.67	63
Antibiotics cost (IV) a day (ICU setting)	$277.50	63
Hospital ward per day ARTIs cost	$2285.00	Calculation
ICU cost per day	$4300.00	64–66
Emergency department cost, including procalcitonin testing	$207	Assumption
Missed bacterial infection, no mortality: +1 hospital day	$2869.88	Assumption/Calculation
Missed bacterial infection, with mortality	$51 680.76	Assumption/Calculation
Missed mortality, no bacterial	$37 730.51	Assumption/Calculation
HostDx Sepsis cost^a^	$0	Assumption

a Costs are unknown, not included in model.

**Table 3:** Expected Costs and Outcomes, SOC (standard of care) vs HostDx Sepsis

	SOC	HostDx Sepsis	Difference (%) SOC vs HostDx Sepsis
Hospital days	2.19	1.38	−0.80 (−36.7%)
Antibiotic days	5.05	3.56	−1.49 (−29.5%)
30-day mortality	12.3%	10.6%	−1.67% (−13.64%)
Total costs (per person)^a^	US$6311	US$4337	US$1974 (−31.3%)
Total costs (1000 cohort)	US$6 311 153	US$4 337 117	US$1 974 036 (−31.3%)

a Model estimates did not include costs of HostDx Sepsis.

**Table 4:** Outcomes Segmented by Ground Truth Patient Status for 1000 Simulated Patients

	Ground Truth			% of Patients in Band	Cost (US$)	Antibiotic Days	Hospital Days	ICU Days	30-day Mortality
	Bacterial Infection	Viral Infection	30-day Mortality						
**Base Case**	No	No	No	26	$1 360 892	1194	473	22	0
	No	No	Yes	3	$473 439	243	166	12	36
	No	Yes	No	32	$1 671 063	1458	579	27	0
	No	Yes	Yes	4	$580 101	295	203	14	44
	Yes	No	No	14	$748 304	709	256	12	0
	Yes	No	Yes	2	$252 944	132	89	6	19
	Yes	Yes	No	17	$915 544	864	313	14	0
	Yes	Yes	Yes	2	$308 866	160	109	7	24
**HostDx Sepsis Case**	No	No	No	26	$724 484	714	235	29	0
	No	No	Yes	3	$498 111	204	160	45	30
	No	Yes	No	32	$926 473	833	298	37	0
	No	Yes	Yes	4	$636 917	253	203	58	38
	Yes	No	No	14	$451 793	596	138	17	0
	Yes	No	Yes	2	$263 627	133	88	19	18
	Yes	Yes	No	17	$530 160	679	160	19	0
	Yes	Yes	Yes	2	$305 552	151	103	22	20
**HostDx Sepsis Case Minus Base Case**	No	No	No		−$636 408	−479	−238	7	0
	No	No	Yes		$24 672	−39	−6	34	−6
	No	Yes	No		−$744 590	−625	−281	10	0
	No	Yes	Yes		$56 816	−42	1	43	−6
	Yes	No	No		−$296 511	−113	−118	5	0
	Yes	No	Yes		$10 683	1	−1	13	−2
	Yes	Yes	No		−$385 384	−185	−153	5	0
	Yes	Yes	Yes		−$3314	−10	−6	15	−3
